# Monkeypox in a Young Infant — Florida, 2022

**DOI:** 10.15585/mmwr.mm7138e3

**Published:** 2022-09-23

**Authors:** Katharine E. Saunders, Andrea N. Van Horn, Helen K. Medlin, Ann Carpenter, Philip A. Lee, Liliana Gutierrez, Joshua Dillon, Alexandra P. Newman, Anne Kimball, David W. McCormick, Danielle R. Stanek

**Affiliations:** ^1^Epidemic Intelligence Service, CDC; ^2^Florida Department of Health; ^3^CDC Monkeypox Emergency Response Team; ^4^Orlando Health, Arnold Palmer Hospital for Children, Orlando, Florida; ^5^AdventHealth for Children, Orlando, Florida; ^6^New York State Department of Health.

In August 2022, the Florida Department of Health (FDOH) was notified of a suspected case of monkeypox in an infant aged <2 months who was admitted to a Florida hospital with a rash and cellulitis. This case report highlights findings from the related epidemiologic investigation and describes the public health actions taken. This activity was reviewed by CDC and was conducted consistent with applicable federal law and CDC policy.* This is the youngest patient with confirmed monkeypox infection in Florida to date.

The infant was initially evaluated in an emergency department (ED) for a raised erythematous rash on the arms, legs, and trunk which had been present for 5 days. A rash swab was collected for bacterial culture and yielded a negative test result. Varicella, herpes simplex virus, and HIV testing were also negative. The patient returned to the ED 2 days later, at which time the rash had progressed to include numerous, diffusely scattered papulovesicular lesions over the body, many with central umbilication. The infant was admitted to the hospital with a diagnosis of molluscum contagiosum and started on intravenous antibiotics for secondary bacterial cellulitis associated with having scratched a lesion on the arm. The lesions subsequently spread to the back, soles of feet, face, and eyelid and became pustular over the first few days of admission. Swabs from forehead and back lesions tested positive for *Orthopoxvirus* DNA and Clade II* Monkeypox virus* DNA by polymerase chain reaction 10 days after rash onset (Figure). Results were confirmed by the Florida public health laboratory and CDC.^†^ FDOH and hospital clinicians consulted with CDC regarding treatment options. The infant was treated with oral tecovirimat and Vaccinia Immune Globulin Intravenous (*1*). Prophylactic trifluridine^§^ drops were administered to prevent ophthalmic complications from the eyelid lesion. The infant remained afebrile and stable throughout the course of illness, tolerated the treatments well, and fully recovered.

**FIGURE:**
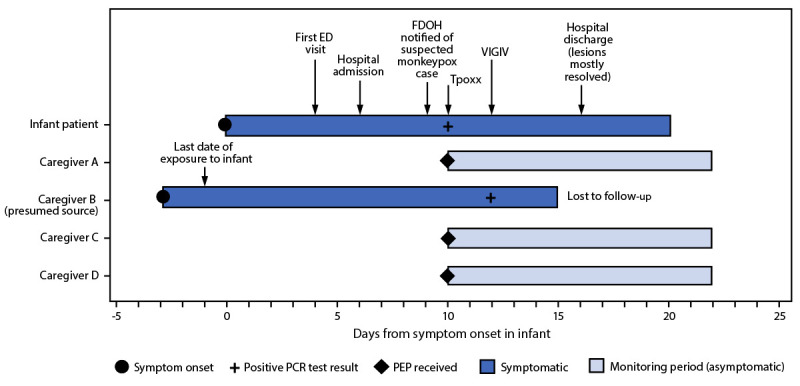
Timeline of symptom onset, testing, treatment, and public health interventions in response to a case of monkeypox in an infant* — Florida, 2022 **Abbreviations**: ED = emergency department; FDOH = Florida Department of Health; PCR = polymerase chain reaction; PEP = postexposure prophylaxis; Tpoxx = tecovirimat; VIGIV = Vaccinia Immune Globulin Intravenous. * Caregiver B and caregiver C shared a bed with infant.

The infant had no history of travel, no history of acute infections in the 3 weeks preceding rash onset, no known immunocompromising conditions, did not attend a child care facility, and had no caregivers outside the home. Within the home, the infant was cared for by four caregivers. Caregiver A acted as the main guardian throughout the infant’s hospital stay and had prolonged exposure with skin-to-skin contact. Caregiver B reported activities that placed him at high risk for monkeypox exposure during the 2 months preceding the infant’s illness (*2*). Caregiver B reported hematuria and fever, followed by a rash within the 3 weeks before the infant’s symptom onset. One day before the infant became symptomatic, caregiver B moved to another state and sought medical care for his symptoms. He received a positive *Orthopoxvirus* DNA test result 2 days after the infant’s positive test result, after which, he was lost to follow-up. The infant had daily close contact with caregiver B in the home for 6 weeks before rash onset. Possible routes of transmission included shared bed linens and skin-to-skin contact through holding and daily care activities. Investigation identified three other household family members with household exposures to both the infant and caregiver B. Caregiver B, caregiver C, and the infant shared a bed for the 6 weeks preceding the infant’s symptom onset. All household members (caregivers A, B, C, and D) held the infant with close skin-to-skin contact. Caregivers A, C, and D received postexposure prophylaxis with JYNNEOS vaccine and remained asymptomatic at 22 days after the infant’s symptom onset (*2*,*3*). Caregiver A had also received smallpox vaccination during childhood.

To date, 27 confirmed cases of monkeypox in pediatric patients aged 0–15 years have been reported in the United States during the 2022 outbreak (*4*). Clinical presentations in children with monkeypox have been similar to those in adults, although children might have a higher risk for severe disease (*5*). Timely laboratory identification and thorough epidemiologic investigation are critical for effective public health response to monkeypox infection. In this case, contact tracing and postexposure prophylaxis vaccination of close contacts of the affected infant might have prevented further transmission to household members (*3*). Clinicians should consider monkeypox infection as a differential diagnosis in pediatric patients with pustular or vesicular rashes and be aware of the possibility for household transmission to pediatric patients, particularly if the children meet epidemiologic exposure criteria for diagnosis of monkeypox (*6*).

## Footnotes

* 45 C.F.R. part 46, 21 C.F.R. part 56; 42 U.S.C. Sect. 241(d); 5 U.S.C. Sect. 552a; 44 U.S.C. Sect. 3501 et seq.

† Patient specimen tested positive for nonvariola* Orthopoxvirus* DNA by polymerase chain reaction at Florida Bureau of Public Health Laboratories and was confirmed positive for Clade II* Monkeypox* *virus* DNA at CDC.

§ Prophylactic trifluridine is an antiviral drug for topical treatment of epithelial keratosis caused by herpes simplex virus.
